# Dehydrogenation Coupling of Methane Using Catalyst-Loaded Proton-Conducting Perovskite Hollow Fiber Membranes

**DOI:** 10.3390/membranes12020191

**Published:** 2022-02-05

**Authors:** Jian Song, Yuepeng Hei, Claudia Li, Naitao Yang, Bo Meng, Xiaoyao Tan, Jaka Sunarso, Shaomin Liu

**Affiliations:** 1 Department of Chemical Engineering, Shandong University of Technology, Zibo 255049, China; Jian.Song@sdut.edu.cn (J.S.); vortecs@163.com (Y.H.); naitao@sdut.edu.cn (N.Y.); mb1963@sdut.edu.cn (B.M.); 2 State Key Laboratory of Separation Membranes and Membrane Processes, Department of Chemical Engineering, Tiangong University, Tianjin 300387, China; 3 Research Centre for Sustainable Technologies, Faculty of Engineering, Computing and Science, Swinburne University of Technology, Jalan Simpang Tiga, Kuching 93350, Malaysia; cli@swinburne.edu.my; 4 Department of Chemical and Biomolecular Engineering, National University of Singapore, Singapore 117585, Singapore; 5 College of Chemical Engineering, Beijing University of Chemical Technology, Beijing 100029, China; Shaomin.Liu@curtin.edu.au

**Keywords:** proton conductor, perovskite membrane, hollow fiber, dehydrogenation coupling of methane (DCM), catalyst loading

## Abstract

Catalytic dehydrogenation coupling of methane (DCM) represents an effective way to convert natural gas to more useful C_2_ products (C_2_H_6_, C_2_H_4_). In this work, BaCe_0.85_Tb_0.05_Co_0.1_O_3−δ_ (BCTCo) perovskite hollow fiber membranes were fabricated by the combined phase inversion and sintering method. SrCe_0.95_Yb_0.05_O_3−δ_ (SCYb) perovskite oxide was loaded as a catalyst onto the inner hollow fiber membrane surface, which promoted the CH_4_ conversion and the C_2_ hydrocarbon selectivity during the DCM reaction. The introduction of steam into the methane feed gas mixture elevated the C_2_ selectivity and yield due to the alleviation of coke deposition. Switching N_2_ to air as the sweep gas further increased the C_2_ selectivity and yield. However, the conversion of methane was limited by both the low permeability of the membrane and the insufficient catalytic activity of the catalyst, leading to low C_2_ yield.

## 1. Introduction

Methane coupling represents an economically promising route to directly convert natural gas feedstock into value-added C_2_ hydrocarbons such as ethane and ethylene [1]. The two routes for methane coupling are oxidative coupling of methane (OCM) and non-oxidative dehydrogenation coupling of methane (DCM) [2]. The presence of oxygen as a reactant in the OCM process presents two severe issues: 1) deep oxidation of C_2_ products, leading to low C_2_ selectivity and yield; and 2) the formation of water byproduct, leading to catalyst deactivation. In contrast, the DCM reaction proceeds without gaseous oxygen (Equations. (1) and (2)):

(1) $$2{\mathrm{CH}}_{4}\Leftrightarrow C_{2}H_{6}+H_{2},{\;\Delta G}_{1000}^{0}=71.1{\;\mathrm{kJ}\;\mathrm{mol}}^{-1}$$

(2) $$2{\mathrm{CH}}_{4}\Leftrightarrow C_{2}H_{4}+2H_{2},{\;\Delta G}_{1000}^{0}=57.2{\;\mathrm{kJ}\;\mathrm{mol}}^{-1}$$

**Figure 1:**
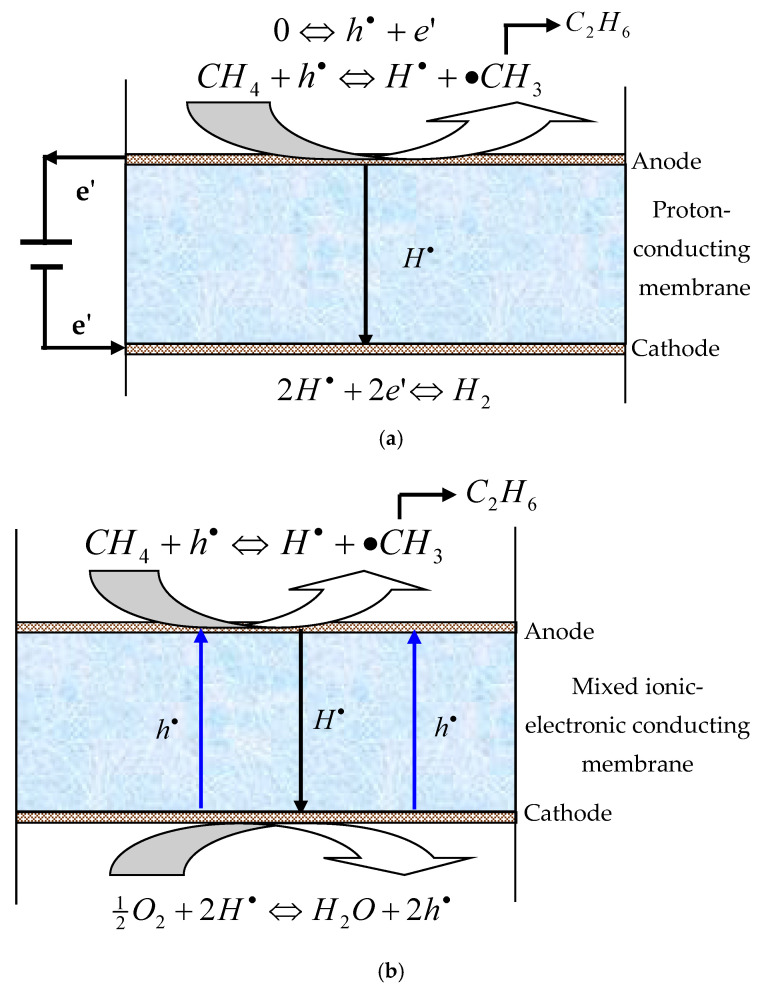
Mechanism of non-oxidative dehydrogenation coupling of methane in the PCMRs (**a**) pumping mode; (**b**) permeation mode.

However, the methane dehydrogenation reactions are thermodynamically unfavorable and display equilibrium-limited conversion. Through the in situ removal of hydrogen from the reaction site using a hydrogen permeable membrane, the equilibrium of the methane coupling reaction can be shifted towards the C_2_ products side, leading to enhanced conversion [3,4]. Figure 1 shows the mechanism of methane coupling in the proton-conducting ceramic membrane reactors (PCMRs). On the anode side, methane is adsorbed and catalytically converted into methyl radicals, which enter the gas phase for coupling reaction. The protons released from the methane coupling reaction are electrochemically driven to the cathode side of the membrane, where they combine with electrons to form hydrogen or react with oxygen to form water.

In addition to the high permselectivity towards hydrogen, the oxide membrane may also serve as an electrolyte to supply or remove ions to or from the catalyst surface, leading to noticeably enhanced catalytic activity and selectivity. Furthermore, compared to the dense metal membranes, the dense ceramic PCMRs possess higher thermal resistance and better chemical stability and are thus more attractive for use in high temperature and harsh environments. However, although dense membranes demonstrate 100% selectivity towards hydrogen permeation, the C_2_ yields are usually very low, which severely limits the practicality of the PCMRs. Iwahara and co-workers [5] introduced the use of electrode and power source in the electrochemical methane coupling reaction. They used a one-end-closed SrCe_0.95_Yb_0.05_O_3−δ_ (SCYb) ceramic tube as a solid electrolyte for the electrochemical reactor, porous silver as the electrode material, and a galvanostat as the power source. Stoukides and co-workers [3] studied the electrocatalytic non-oxidative dimerization of CH_4_ to C_2_H_4_ and C_2_H_6_ in a continuous stirred tank reactor at 600–750 °C and in atmospheric total pressure using an SCYb electrolyte. They reported that the presence of H_2_O or O_2_ increased the protonic conductivity of the electrolyte and the reaction rate of CH_4_ dehydrogenation to C_2_H_4_ and C_2_H_6_ was enhanced compared with the open-circuit reference case. In addition, the electrochemical attempts, a non-electrochemical approach was also reported to catalyze CH_4_ pyrolysis to form C_2_ hydrocarbons and H_2_ on one side, while hydrogen was transported through a proton conducting membrane to the other side without the need of electrode or power supply [6].When the membrane is a mixed proton-electron conductor, the hydrogen can be extracted from the reaction system using the self-discharge phenomenon. In this case, the external electric source, electrode materials and current collectors are unnecessary to transport protons across the solid electrolyte and the construction of the reactor is more simplified (permeation mode, Figure 1b). However, the methane activation and dimerization rate may be many times lower than that of the pumping operation mode [3,5]. 

The catalytic activity of membranes is of crucial importance for the OCM reaction in dense ceramic membrane reactors [7,8], which is also applicable for the DCM reaction. However, it is difficult to design a membrane composition with both high catalytic activity and high hydrogen permeability. Furthermore, the exposure of catalytically modified membrane surfaces can also be maximized in this case for higher methane conversion. In fact, for the DCM reaction, a high C_2_ yield can be conditionally achieved when the hydrogen permeation flux, methane flow rate and intrinsic reaction rate match each other well, which is similar to the conditions for OCM reaction [8]. 

Hollow fiber membranes fabricated via the phase inversion/sintering technique can be used to obtain high oxygen permeation fluxes due to their asymmetric structure (i.e., a thin dense separation layer integrated with a porous substrate of the same material) [9,10,11,12,13]. Furthermore, such hollow fiber membranes also possess large membrane surface area to reactor volume ratios, which favor the high C_2_ yields [14,15]. Hence, mixed proton-electron/hole conducting hollow fiber membranes fabricated through the same technique can function as hydrogen separation membranes for DCM reaction. To obtain high C_2_ yield, catalysts are usually applied to activate the methane dissociation and hydrogen combination reactions, of which such catalysts may be of Ag, Ni or other specially designed oxides [5,16,17]. It is worth noting that several mixed conducting perovskites (e.g., strontium cerates) are very efficient methane coupling catalysts and have been reported as catalytic reactors [6,18,19].

In this study, BaCe_0.85_Tb_0.05_Co_0.1_O_3−δ_ (BCTCo) perovskite hollow fiber membranes with a desired structure were prepared by the phase inversion/sintering technique. SrCe_0.95_Yb_0.05_O_3−δ_ (SCYb) perovskite oxide was loaded on the inner surface of the BCTCo hollow fibers as the DCM catalyst. The SCYb oxide was reported to exhibit good DCM catalytic activity due to its high proton and electron-hole conductivities that favor the enhanced formation of C_2_ hydrocarbons while reducing that of CO_x_. 

## 2. Experimental

### 2.1. Preparation of the Perovskite Powders and Hollow Fiber Membranes

BaCe_0.85_Tb_0.05_Co_0.1_O_3−δ_ (BCTCo) perovskite was used as the hydrogen permeable membrane material, while SrCe_0.95_Yb_0.05_O_3−δ_ (SCYb, purchased from PRAXAIR, Danbury, CT, USA) was used as the DCM catalyst. The perovskite oxides were prepared by a EDTA-citric acid assisted sol-gel combustion method as described elsewhere [20]. Metal nitrates such as Ba(NO_3_)_2_, Ce(NO_3_)_3_·6H_2_O, Tb(NO_3_)_3_·6H_2_O, and Co(NO_3_)_2_·6H_2_O were used as the raw materials for metal ion sources. Calcination of the composite oxide precursors was conducted at 800 °C in static air atmosphere for 4 h to remove the residual carbon and to form the desired perovskite structure.

To prepare the BCTCo hollow fiber membranes using the phase inversion/sintering technique, the BCTCo powder was ball-milled for 10 h and then sieved using a 200-mesh sifter to remove agglomerates. The detailed preparation procedures were described elsewhere [9]. In this study, the spinning suspension consisted of 66.67 wt% BCTCo powders, 6.67 wt% polysulphone (PSU) (Udel^®^ P3500, Solvay, Brussels, Belgium) as the polymer binder and 26.67 wt% 1-methyl-2-pyrrolidinone (NMP) (AR Grade, >99.8%) as the solvent. A spinneret with an orifice diameter/inner diameter of 3.0/1.5 mm was used to spin the hollow fiber precursors. DI water and tap water were used as the internal and external coagulants, respectively. After drying and straightening, the hollow fiber precursors were sintered at 1350 °C for 4 h to form dense hollow fiber membranes. The gas tightness of the hollow fibers for subsequent hydrogen permeation tests and surface loading was measured by nitrogen gas permeation test [10].

### 2.2. Catalyst Modification of the Hollow Fiber Membranes

To coat the DCM catalyst layer on the inner surface of the BCTCo hollow fibers, a catalyst slurry was prepared by mixing the SCYb perovskite powders in ethanol followed by grinding in an agate mortar and pestle for 10 min. PVP-K30 (30 mg g^−1^ perovskite) and polyvinyl butyral (40 mg g^−1^ perovskite) were used as additives and added into the slurry followed by another 10 min of grinding. The hollow fiber lumen was perfused with the homogenous slurry to deposit the perovskite on the inner surface followed by sweeping with Ar to blow out the extra solution. The coated fibers were then subjected to the post-heat treatment with a ramp rate of 5 °C min^−1^ to 600 °C, held for 1 h to remove the organic species and then sintered at 1050 °C in stagnant air for 2 h (ramp rate 2 °C min^−1^) to integrate the coating layer onto the membrane surface. Finally, the furnace was cooled down to room temperature at 2 °C min^−1^. To obtain a thicker perovskite layer, the coating/sintering operation was repeated for 2–3 times. As for the packing comparison, a small amount of SCYb catalyst powder was packed on the inner surface of the middle section of the hollow fiber, which was within the effective heating zone of the furnace. 

### 2.3. Hydrogen Permeation and DCM Reaction in the Hollow Fiber Membranes 

**Figure 2:**
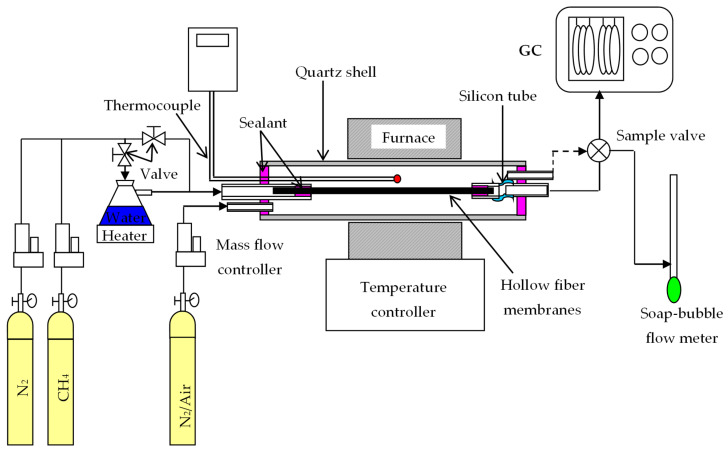
Experimental setup for DCM reaction.

A BCTCo hollow fiber membrane with a typical length of 300 mm, which was tested for gas tightness by N_2_ permeation, was assembled in a quartz tube with external/internal diameter of 10/8 mm and a length of 500 mm. A flexible silicone rubber tube was used on one end of the fiber to offset the thermal expansion mismatch between the hollow fiber membrane and the module shell at high temperatures, as shown in Figure 2. The sealing was achieved using an organic sealant. A K-type thermocouple was positioned close to the middle section of the hollow fiber to measure the temperature during operation. The membrane reactor was assembled using either a blank membrane (a membrane without catalyst loading) or a catalytic membrane (membrane with coated/packed catalyst) and placed in a custom-made tubular furnace with a constant heating length of 5 cm. The permeation equation through the BCTCo hollow fiber membrane can be expressed as [20]:

(3) $$J_{H_{2}}=\frac{V_{t}}{A_{m}}\left(y_{H_{2}}-y_{He}\;\right)$$

where *V_t_* is the flow rate of the permeate stream; $y_{H_{2}}$ and $y_{He}$ are the hydrogen and helium fractions in the permeated stream, respectively; *A_m_* is the effective membrane area for hydrogen permeation, which is calculated by

(4) $$A_{m}=\frac{\pi \left(d_{out}-d_{in}\right)L_{e}}{\ln(d_{out}/d_{in})}$$

in which *L_e_* is the effective fiber length for hydrogen permeation (5 cm in this work); $d_{out}$ and $d_{in}$ are the outer and inner diameter of the hollow fiber membrane, respectively. The helium concentration is present in Equation (3) if a minor leakage occurs during the permeation and thus, the amount of leaked hydrogen should be deducted to obtain the net permeation flux. It is also worth noting that the hydrogen permeation beyond the central heating zone was not considered since its contribution towards the overall hydrogen flux is much lower than the central part.

For the DCM operation, dry or steam-saturated CH_4_ diluted by N_2_ was introduced into the test cell and passed onto the fiber lumen while N_2_ or air was flown concurrently over the shell side of the membrane reactor. Steam along with CH_4_ and N_2_ was fed to the hollow fiber lumen to retard the coke deposition rate, thereby inhibiting the catalyst deactivation. Air was introduced to the shell side of the BCTCo hollow fibers to further improve the hydrogen permeation through the hollow fiber membranes. The gas flow rates were controlled using mass flow controllers (D07-7B, Beijing Sevenstar Electronics Co. Ltd., Beijing, China), which were calibrated to standard conditions using a soap bubble flow meter. The effluent flow rates were also measured by the soap bubble flow meter. All gas flows were quoted at standard temperature and pressure (STP). The compositions of the exhaust gas were analyzed by a gas chromatograph (Agilent 6890N, Santa Clara, CA, USA) equipped with a 5A molecular sieve column (3 m × Φ2.1 mm), a Porapak Q column (3 m × Φ2.1 mm), TCD and FID detectors. High purity argon (purity ≥99.999%) and hydrogen (purity ≥ 99.999%) was used as the carrier gas with a flow rate of 30 mL min^−1^. Three analyses were conducted for each experimental condition. The methane conversion, C_2_ species selectivity and yield were calculated according to Equations (5) to (7), respectively.

(5) $$X_{CH4}=\left(1-\frac{F_{out}x_{CH_{4}}}{F_{in}y_{f}}\right)\times 100\%$$

(6) $$S_{C_{2}}=\frac{2F_{out}x_{C_{2}}}{F_{in}y_{f}-F_{out}x_{CH_{4}}}\times 100\%$$

(7) $$Y_{C_{2}}=\frac{2F_{out}x_{C_{2}}}{F_{in}y_{f}}\times 100\%$$

where *F_in_* and *F_out_* are the flow rates of methane feed (methane-nitrogen mixture) and product stream, respectively; $y_{f}$ and $x_{C_{2}}$ are the methane concentration in feed and the C_2_ (C_2_H_4_ + C_2_H_6_) concentration in the product stream, respectively. With the methane feed and the product concentrations, the carbon balance could also be calculated to show the coking deposition in the membrane reactor.

### 2.4. Characterization

The morphology of the hollow fiber membranes was observed using a scanning electron microscope (SEM, Hitachi S-4800, Tokyo, Japan). Gold sputter coating was conducted on the sample surface under vacuum before the measurements. The crystal structure of the powders was identified by X-ray diffractometer (Bruker D8 Advance, Karlsruhe, Germany) using Cu-Kα radiation. Continuous scan mode was used to collect 2θ data from 20° to 80° with a 0.02° sampling pitch and a 2° min^−1^ scan rate. The X-ray tube voltage and current were set at 40 kV and 30 mA, respectively.

## 3. Results and Discussion

### 3.1. Crystalline Phase Structure of the Powders and the Membrane

**Figure 3:**
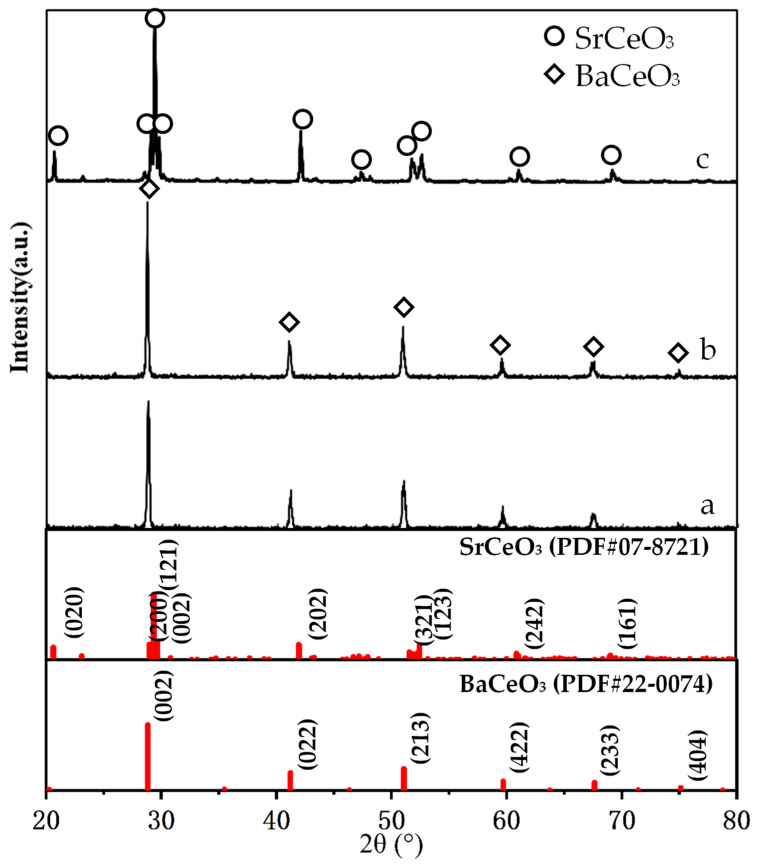
Powder XRD patterns of the (**a**) BCTCo powder, (**b**) the BCTCo hollow fiber membrane and (**c**) SCYb powder.

Figure 3 shows the XRD patterns of the BCTCo powder, hollow fiber membrane, and the SCYb powder. The pure perovskite phase structure formed successfully after the BCTCo powder precursors were calcined at 800 °C for 4 h (Figure 3a). For comparison, the XRD pattern of the BCTCo hollow fiber membranes after a heat treatment of 1350 °C for 4 h is also depicted in Figure 3b. No changes were observed in the crystalline structure of BCTCo after the powders were fabricated into hollow fiber membranes by the spinning and sintering processes, which indicates that the perovskite structure was perfectly preserved during the spinning and sintering process. However, the corresponding characteristic peaks intensity of the perovskite phase in the sintered BCTCo hollow fibers are slightly higher than those in the original BCTCo powders. Using Jade 5.0 software, the crystallites sizes for BCTCo pure powder and hollow fibers are calculated to be 429 (184) Å and 1266 (179) Å, respectively. This suggests that the crystal size in the hollow fibers increased due to the high-temperature sintering. The crystalline structure of SCYb oxides was plotted in Figure 3c, the XRD pattern exhibits seven strong diffraction peaks with corresponding 2θ angles of 20.6, 29.4, 42.1, 51.9, 52.6, 61.0 and 69.1, which have previously been reported to correspond to the orthorhombic perovskite phase of SCYb [21,22].

### 3.2. Morphology of the Hollow Fiber Membranes

**Figure 4:**
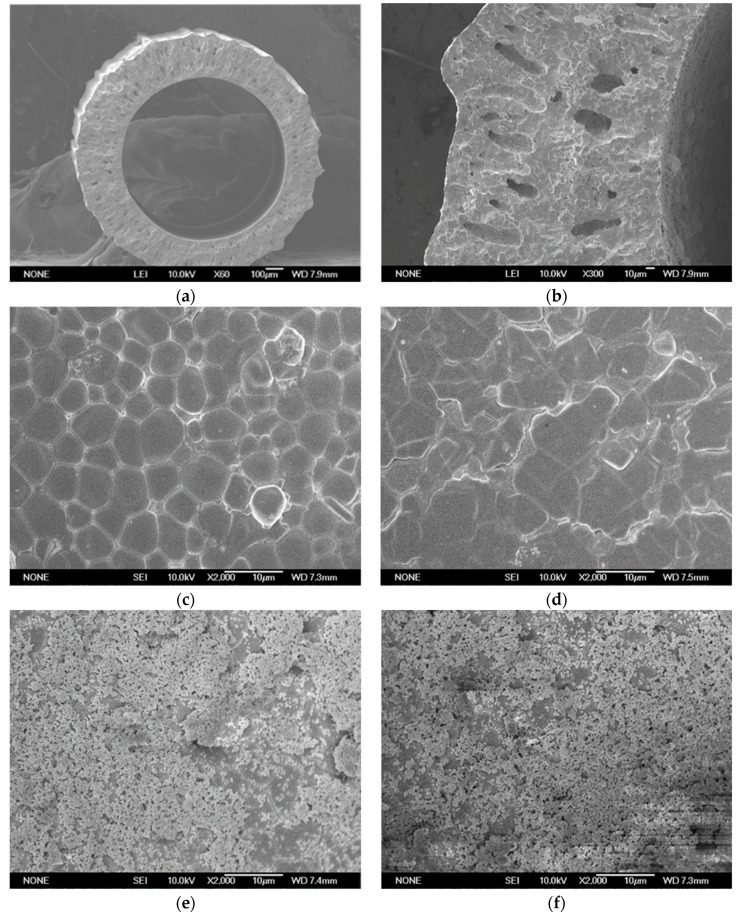
SEM micrographs of the BCTCo hollow fiber membranes: (**a**) cross-section with no catalyst loaded; (**b**) cross-section with SCYb oxide coated once; (**c**) inner and (**d**) outer surface with no catalyst loaded; inner surface coated with SCYb oxide (**e**) before and (**f**) after DCM reaction.

Figure 4 shows the SEM microstructure of BCTCo hollow fiber membrane prepared by the phase-inversion/sintering technique. The outer and inner diameter of the hollow fiber membrane is approximately 1.38 mm and 0.93 mm, respectively (Figure 4a). Despite the presence of numerous pores on the inner hollow fiber membrane wall, both the inner and outer surfaces are dense as confirmed by the subsequent nitrogen permeation test. Figure 4b shows the thickness of the SCYb catalyst coating layer is about 3 μm, which is coated through a single operation on the inner surface of the hollow fiber. Figure 4c,d show the internal and external surfaces of the hollow fiber membrane, respectively, both of which are quite dense and smooth. Figure 4e demonstrates the fiber membrane inner surface after loading the SCYb catalyst. As illustrated, the catalyst loaded through a single coating operation is sufficient to cover the surface of the fiber membrane. Several discrete holes (Figure 4e) were produced by the combustion of added organic additives during the calcination process, which can effectively increase the hydrogen flux. During the direct coupling of methane, the inner hollow fiber surface was exposed to reducing atmosphere and the outer surface was exposed to a neutral or oxidizing atmosphere. Through coupling reaction of methane under reducing atmosphere, which can be seen from Figure 4f, the perovskite particles were broken down into smaller grains. This tallies with the similar observation and detailed analysis by Tsai et al. [23] during the production of syngas using a ceramic membrane.

### 3.3. Hydrogen Permeation in the Uncoated BCTCo Hollow Fiber Membrane

**Figure 5:**
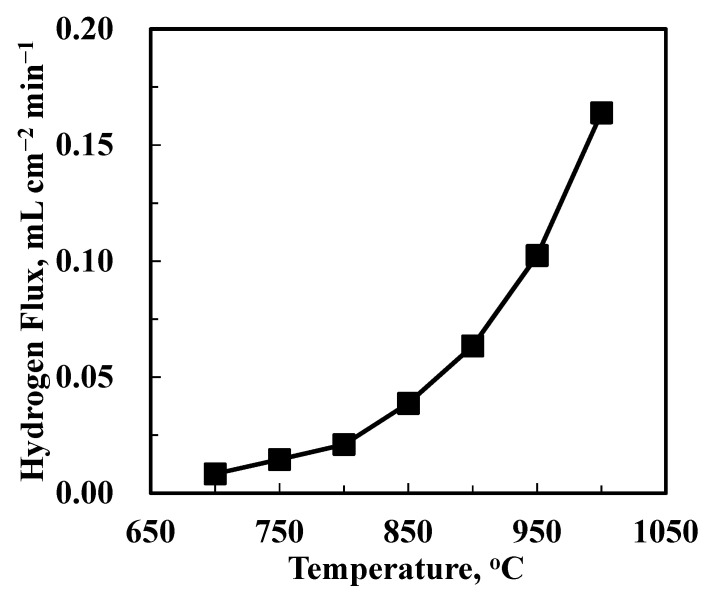
Hydrogen permeation through the BCTCo hollow fiber membrane with no catalyst loaded under H_2_-He/N_2_ gradient at different temperatures.

The hydrogen permeation performance of the uncoated BCTCo hollow fiber membrane was measured with N_2_ fed into the hollow fiber lumen, while H_2_-He mixture was flown over the outer surface of the fiber. Figure 5 shows the experimental results of hydrogen permeation flux while the H_2_-He feed flow rate was fixed at 15–15 mL min^−1^ and N_2_ flow rate was fixed at 30 mL min^−1^. As expected, the hydrogen permeation rate increased with increasing temperature, i.e., from 0.0112 mL cm^−2^ min^−1^ at 700 °C to 0.167 mL cm^−2^ min^−1^ at 1000 °C, since both the surface exchange reaction and the ionic bulk diffusion are temperature-activated. This leads to an increment in the hydrogen permeate concentration, which implies that the operating temperature plays a more important role than the pressure driving force in hydrogen permeation through the perovskite membranes.

### 3.4. DCM Reaction in the Catalyst Loaded BCTCo Hollow Fiber Membranes

Regardless of the form of oxygen introduced into the reaction chamber of OCM membrane reactors, the introduction of O_2_ decreases the inherent C_2_ product selectivity and increases the cost incurred for the reaction due to the formation of carbon oxides and water. Hence, methane coupling can also occur through non-oxidative methods where a hydrogen permeable membrane reactor is used while the usage of oxygen can be avoided. It is well known that dehydrogenation reactions (Equations (1) and (2)) can occur at elevated temperature. However, these reactions are thermodynamically unfavorable and limited by the equilibrium conversion. This disadvantage can be overcome by using a suitable membrane reactor according to the non-oxidative dehydrogenation coupling of methane mechanism shown in Figure 1. By transferring hydrogen from the methane side to the other side, the equilibrium of the methane coupling reaction may be driven towards ethane and higher reactant conversion. Based on this concept, catalytic membrane reactors have been widely explored to study the methane coupling reaction [4,5,24,25,26,27,28]. The results of some typical experimental results of the methane coupling in PCMRs are summarized in Table 1. Hamakawa et al. [5,27] reported the use of mixed conducting SrCe_0.95_Yb_0.05_O_2.95_ ceramic membranes with or without porous Ag electrodes for the DCM reaction and hydrogen permeation studies. They concluded that in hydrogen atmosphere, the membrane would transform from a proton conductor to a mixed proton-hole conductor, which allows hydrogen to permeate without the use of electrodes or an external power source. The C_2_ product yield was enhanced from 0.23 to 1.33 µmol min^−1^ cm^−2^ through the continuous removal of the hydrogen formed from the reaction zone with a selectivity of nearly 100% (reaction temperature of 900 °C and membrane thickness of 1 mm).

In another work featuring mixed proton-hole conducting BaCe_0.95_Mn_0.05_O_3−y_ membrane (reaction temperature of 950 °C and membrane thickness of 1 mm), a C_2_ product yield of 8.9 µmol min^−1^ cm^−2^ was obtained according to White et al. [28]. However, most of the reported conversion and C_2_ yields remained too low (less than 1% and 2%, respectively, as shown in Table 1) and coke was formed during the experiment. The inability to obtain a higher C_2_ yield in these membrane reactors was linked to the inherent problems of the membrane reactors such as the low catalytic activity of the membrane surface and catalyst, the high membrane thickness, the occurrence of methane pyrolysis in the absence of oxygen, low oxygen fluxes in the DCM reaction temperature range, i.e., 700–1000 °C and unfavorable reactor configuration [7].

**Figure 6:**
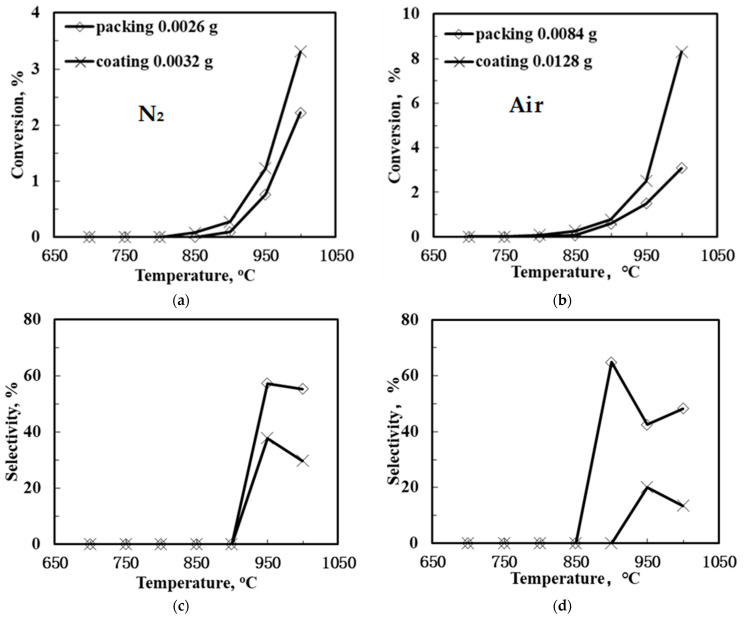

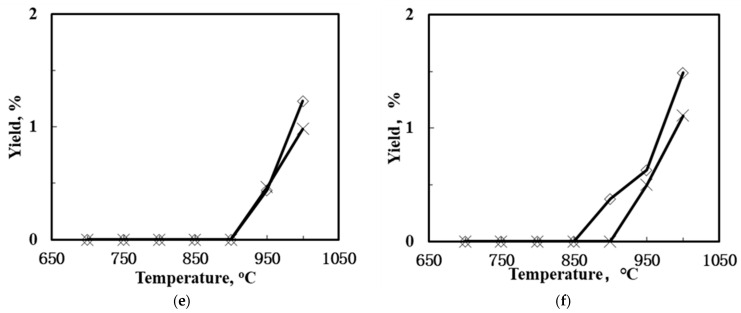
CH_4_ conversion (**a**,**b**), C_2_ selectivity (**c**,**d**) and C_2_ yield (**e**,**f**) of the SCYb-loaded BCTCo hollow fiber membrane reactors against temperature under N_2_ (left) and air (right) sweep gas (Methane steam feed flow rate = 30 mL min^−1^, methane feed concentration = 33%, N_2_ or air sweep flow rate = 40 mL min^−1^).

Figure 6 shows the methane conversion and C_2_ product selectivity and yield of the SCYb-loaded BCTCo hollow fiber membranes at different temperature when N_2_-diluted CH_4_ was flowed through the fiber lumen. N_2_ or air functioned as the sweep gas on the outer membrane surface. Evidently, when the sweep gas was changed from N_2_ to air, both the CH_4_ conversion and C_2_-yield were raised to different extent. The SCYb-loaded BCTCo hollow fiber membrane reactor operated at 1000 °C with N_2_ sweep gas resulted in CH_4_ conversion (Figure 6a) and C_2_-hydrocarbons yield (Figure 6e) of 2.22% and 1.23%, respectively, while 3.08% CH_4_ conversion (Figure 6b) and 1.49% C_2_ product yield (Figure 6f) was obtained when air was used as the sweep gas. This increase is attributed to the oxygen permeation performance of the BCTCo hollow fiber membrane [20]. At elevated temperature, oxygen in air permeates through the membrane bulk to the fiber lumen and reacts with methane, which promotes the rate of methane conversion. However, based on the gaseous radical reaction mechanism [7,8,32,33,34]: methane is first adsorbed and reacts with the lattice oxygen ($O_{O}^{x}$) to form methyl radicals, which are then coupled in gas phase into ethane or further reacted with gaseous oxygen to form carbon oxides, while ethylene is formed by further dehydrogenation of ethane on the membrane surface. It is believed that the generation of methyl radicals is the rate limiting step and it is also commonly agreed that the OCM undergoes the dissociation of the methane into methyl radical at the active sites on the catalyst surface. These active sites are usually reactive oxygen (O^−^, O_2_^2−^) and lattice oxygen species (O^2−^). Hence, although some lattice oxygen ($O_{O}^{x}$) can react with methane to form methyl radicals that are later coupled in gas phase to form ethane, a small portion of methane will further react with gaseous oxygen to form carbon oxides. Simultaneously, the generated H_2_, C_2_-hydrocarbons and coke will be further oxidized into H_2_O and CO_x_ under the high temperature, which will reduce the C_2_-hydrocarbons selectivity and is not conducive to the generation of the target products.

Nevertheless, the C_2_-hydrocarbons yield was increased in varying degrees due to the enhanced CH_4_ conversion and the catalytic effect of the permeated oxygen. On the other hand, the protons released from the methane coupling reaction are permeated to the shell side of the membrane under the hydrogen pressure gradient, where they react with oxygen to form water. With the consistent removal of hydrogen from the reaction site using the proton conducting membrane, the equilibrium of the methane coupling is driven towards the C_2_ product side, leading to enhanced conversion [3,4].

An interesting observation from this study is that the packed membrane reactor performed better compared to the corresponding SCYb-coated membrane. For instance, when steam was introduced into the feed gas and N_2_ was swept over the outer hollow fiber surface at 1000 °C, the CH_4_ conversion and C_2_ product yield was 4.54% and 1.86%, respectively, for the packed membrane reactor, while only 4.16% CH_4_ conversion and 1.42% C_2_ product yield was obtained for the coated membrane. This can be attributed to the location of the packed catalyst that was in the middle of the heating reaction zone, which fully contributed towards the reaction. In contrast, although the inner hollow fiber surface was entirely coated with the SCYb catalyst, only the area within the effective reaction zone was utilized in the catalytic reaction, which comprised merely 16% of the coated catalyst. Furthermore, the packed catalyst was in the form of microspheres, which presented a large specific surface area due to the tiny particle size and loose deposits. However, when the coated catalyst was sintered to the surface of fiber membrane wall, the grain size grew larger and the specific surface area decreased. This led to a reduction in the available area for reaction, which affected the catalyst performance.

**Figure 7:**
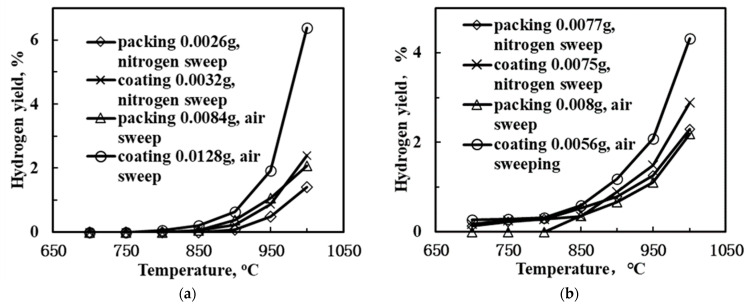
Hydrogen yield of the SCYb-loaded BCTCo hollow fiber membrane reactor with different catalyst loading method and sweep gas species: methane feed (**a**) without and (**b**) with steam.

Figure 7 displays the H_2_-yield as a function of temperature in the SCYb-loaded BCTCo hollow fiber membranes. When N_2_ was used as the sweep gas and steam was introduced into the methane gas mixture, the hydrogen yield was promoted compared to the configuration without the use of steam, regardless of whether the catalyst was packed or coated on the membrane inner surface. This was attributed to the enhancement of methane conversion caused by the introduction of steam and the decomposition of steam into hydrogen and oxygen, which directly increased the hydrogen production. According to Oklany et al. [35] and Hou and Hughes [36], the increase in steam used in the methane steam reforming reaction favors increased conversion of methane. The addition of steam to the reactants resulted in the enhanced formation of hydrogen and a portion of the OH radicals generated from the decomposition of steam were consumed by the hydrogen abstraction from the CH_4_, resulting in CH_3_ radicals and water [37]. In addition, the strategy for coating the catalyst appeared to be more convenient for the hydrogen production compared to packing the catalyst. Following calcination, the coated catalyst adhered firmly to the inner surface of the BCTCo hollow fiber membrane. The protons generated from the DCM reaction and the decomposition of steam were transferred swiftly from the catalyst to the membrane and permeated to the membrane shell side, which further increased the hydrogen yield. As for the membrane reactor with catalyst packed inside the hollow fiber, the protons that were initially produced from the DCM reaction were combined with hydrogen molecules and then decomposed into protons on the inner hollow fiber membrane surface. This further complicated the reaction process and decreased the hydrogen permeation performance, thus inhibiting the hydrogen yield. For instance, at 950 °C, the hydrogen yield of the coated membrane reactor with steam introduced into the CH_4_ feed mixture reached 1.48% while only 0.87% yield was obtained for the reactor without steam. Correspondingly, with the introduction of steam at 900 °C, hydrogen yield of 0.9% and 0.78% were obtained for the coated and packed membrane reactor, respectively. Similar trends were observed when the sweep gas was changed from N_2_ to air. In other words, when air was used as the sweep gas on the shell side, the hydrogen yield was also improved compared to the use of N_2_ sweep. An increase in temperature while using air as the sweep gas resulted in the permeation of hydrogen to the air side through the membrane, whereby it would react with oxygen. This caused a reduction in the hydrogen pressure on the lean hydrogen side, thus increasing the transmembrane pressure and permeation driving force of hydrogen. Simultaneously, oxygen would permeate to the methane reaction side, catalyze the DCM reaction and react with the formed protons to consequently elevate the hydrogen yield. For example, when steam was mixed into the methane feed gas of the SCYb-coated BCTCo membrane reactor at 1000 °C, a hydrogen yield of 4.32% was achieved when air was used as the sweep gas, while only 2.89% hydrogen yield was obtained when N_2_ sweep gas was used.

**Figure 8:**
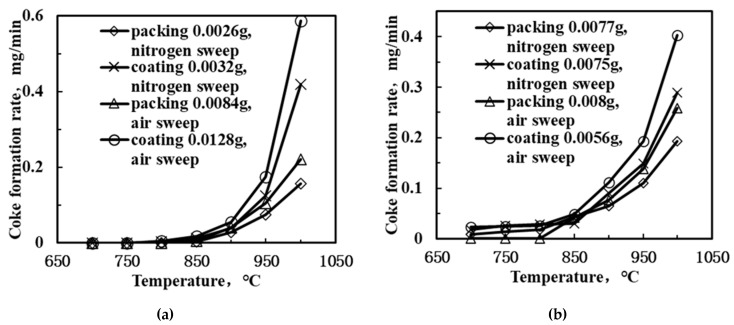
Coke formation rates as a function of temperature for DCM reaction: Methane feed (**a**) without and (**b**) with steam.

Figure 8 shows the calculated coke yields as a function of operating temperature in the DCM membrane reactor. The resultant trend of the coke yield is similar to that of the hydrogen production as previously exhibited in Figure 7. Based on several reports of prior studies [38,39,40,41], some of the generated carbon was removed by steam to form CO and H_2_, or by oxygen and the generated CO_2_ to form CO. Accordingly, the principal methane reactions along with their Gibbs free energies and enthalpy change can be described according to Equations (8)–(11):

CH_4_ → C + 2H_2_; △G_1023K_ = −22 kJ (8)

H_2_O + C → CO + H_2_; △G1023K = −11 kJ, △H1073K = 135.9 kJ mol^−1^ (9)

O_2_ + C → CO_2_; △H_1073K_ = −394.7 kJ mol^−1^ (10)

CO_2_ + C → 2CO; △H_1073K_ = 174.5 kJ mol^−1^ (11)

During coke elimination, the surface carbon was removed and the surface area of the membrane reactor was restored, which helped to preserve further catalytic activity of the membrane. However, although a certain amount of coke was eliminated, the coke formation rate was relatively higher compared to the elimination rate, which resulted in the net accumulation of coke. Nevertheless, despite the promotion of the coke elimination, the introduction of steam or air also resulted in the acceleration of the methane conversion, which led to the facilitation of coke formation. Consequently, the coke formation rate was elevated for the PCMRs with steam or air introduced compared with the PCMRs without the introduction of steam or those using nitrogen as the sweep gas. Another interesting phenomenon is that the coke formation rate for the catalyst-coated PCMRs is higher as compared to the catalyst-packed PCMR, which is in accordance with the rate of hydrogen production shown in Figure 7. The reason for this phenomenon may be linked to the accelerating effect of the catalyst coat for proton conduction, which increased the hydrogen transport rate across the membrane. Hence, the reaction is shifted forward to the C_2_ products side as more methane takes part in the reaction, although the rate of coke formation is simultaneously accelerated.

## 4. Conclusions

BaCe_0.85_Tb_0.05_Co_0.1_O_3−δ_ (BCTCo) hollow fiber membrane was prepared though a combined phase-inversion and sintering method with BCTCo perovskite powder as the raw ceramic materials. SrCe_0.95_Yb_0.05_O_3−δ_ (SCYb) oxide was used as the catalyst for the direct coupling of methane (DCM) reaction. The SCYb-loaded BCTCo membrane reactors were assembled and their DCM properties were analyzed. The catalyst loading method, presence of steam in the feed gas, and type of sweep gas in the membrane shell side collectively contributed towards the DCM performance of the membrane reactor. Accordingly, SCYb-packed membrane reactor performed better relative to the SCYb-coated membrane counterpart due to the larger specific surface area. The use of steam in the methane feed gas mixture could mitigate the coke accumulation and improve the methane conversion and C_2_-hydrocarbons selectivity and yield. When air was swept over the membrane reactor shell side, the performance of the membrane reactor was also promoted relative to when nitrogen was used as the sweep gas. Although the BCTCo membrane reactor generally displays good C_2_ selectivity and hydrogen production rate, the conversion of methane is relatively low, leading to an inadequate C_2_ yield. Overall, the DCM performance of the membrane reactor is largely dependent on the catalytic properties. Hence, more attention needs to be focused on more effective DCM catalyst development to further increase the methane conversion and C_2_-hydrocarbons yield.

## Figures and Tables

**Table 1:** Dehydrogenation coupling of methane in PCMRs.

Membrane (Material, Type and Thickness)	Catalyst	Reaction Temperature (°C)	Max. C_2_ Yield (%)	Study TYPE ^a^	Notes	Ref.
SCYb, disk, 1 mm	Ag	900	0.15	I	S_max_: 100%	[3,5]
SCYb, disk, 1 mm	Ag	900	0.54	I and III	S_max_: 100%	[5]
SCYb, hollow fiber		950	13.4	III	S_max_: 21%	[6]
SCYb, disk, 0.75 mm	Ag	750	0.17	I	C_2_ yield could increase as much as 8 times compared to the open-circuit mode	[25,29]
SCYb, disk, 1 mm	Ag	900	0.06	III	S_max_: 100%; Y_max_: 1.33 µmol·min^−1^·cm^−2^	[27]
BCM, cylindrical, 1 mm	N/A	950	-	III	S_max_: 100%; Y_max_: 8.9 µmol·min^−1^·cm^−2^	[28]
SCYb, disk, 1.5mm	Ag	750	0.55	I	S_max_: 64%	[30]
SCYb, disk, 0.5 mm	Pt	1000	-	II	Trace of C_2_ product observed with electric current range from 20 to 240 mA	[31]
BCTCo, hollow fiber, 224 µm	SCYb	1000	2.61	III	S_max_: 81.13%; Y_max_: 19.66 µmol·min^−1^·cm^−2^	This work

^a^ I, II and III represent H_2_ pump, methane fuel cell for cogeneration of electric current and C_2_ product and mixed proton and hole conduction, respectively. SCYb = SrCe_0.95_Yb_0.05_O_3−δ_; BCM=BaCe_0.95_Mn_0.05_O_3−δ_; S_max_: Maximum selectivity; Y_max_: Maximum C_2_ yield.

## Data Availability

The data presented in this study are available on request from the corresponding author.
